# Microglial modulation as a therapeutic strategy in Alzheimer's disease: Focus on microglial preconditioning approaches

**DOI:** 10.1111/jcmm.18554

**Published:** 2024-08-05

**Authors:** Younes Yassaghi, Yasaman Nazerian, Mobina Ghasemi, Amirhossein Nazerian, Fatemeh Sayehmiri, George Perry, Hamid Gholami Pourbadie

**Affiliations:** ^1^ School of Medicine Shahid Beheshti University of Medical Sciences Tehran Iran; ^2^ School of Medicine Iran University of Medical Sciences Tehran Iran; ^3^ Skull Base Research Center, Loghman Hakim Hospital, Shahid Beheshti University of Medical Sciences Tehran Iran; ^4^ Department of Neuroscience, Development, and Regenerative Biology University of Texas at San Antonio San Antonio Texas USA; ^5^ Department of Physiology and Pharmacology Pasteur Institute of Iran Tehran Iran

**Keywords:** Alzheimer's disease, microglia, neurodegeneration, Neuroinflammation, preconditioning

## Abstract

Alzheimer's disease (AD) is a progressive disease that causes an impairment of learning and memory. Despite the highly complex pathogenesis of AD, amyloid beta (Aβ) deposition and neurofibrillary tangles (NFTs) formation are the main hallmarks of AD. Neuroinflammation also has a crucial role in the development of AD. As the central nervous system's innate immune cells, microglial cells are activated in AD and induce inflammation by producing pro‐inflammatory mediators. However, microglial activation is not always deleterious. M2‐activated microglial cells are considered anti‐inflammatory cells, which develop neuroprotection. Various approaches are proposed for managing AD, yet no effective therapy is available for this disorder. Considering the potential protective role of M2 microglia in neurodegenerative disorders and the improvement of these disorders by preconditioning approaches, it can be suggested that preconditioning of microglial cells may be beneficial for managing AD progression. Therefore, this study review microglial preconditioning approaches for preventing and improving AD.

## 1 INTRODUCTION

Alzheimer's disease (AD) is a progressive neurodegenerative disease impairing cognitive function and causing behavioural changes. This debilitating disease is the most common form of dementia, according to World Health Organization (WHO)'s report on September 2022. It is the seventh cause of death worldwide, leading to disability and dependency in the elderly. Despite various proposed prevention and treatment approaches, these approaches were not satisfying for AD treatment.^1^ The exact cause of AD is still under debate; however, two classic hypotheses for its pathophysiology are extraneuronal deposition of amyloid‐β (Aβ) proteins^2^ and intra‐neuronal neurofibrillary tangles (NFTs),^3^ both of which are main hallmarks of AD. In addition, the role of neuroinflammation and microglia has been identified in AD's pathogenesis by pro‐inflammatory cytokines in brain tissues^4^ and microglia activation by sAPP‐α.^5^ Also, studies have shown the relationship between microglial activation and Aβ and tau proteins^6^, ^7^, ^8^ and suggested that microglia acts as a bridge between Aβ and tau in AD.^9^

Microglial cells are proposed as the central nervous system's innate immune cells with a mesodermal origin,^10^ which in physiological conditions, survey and maintain the function of neurons and synapses.^11^ Through stimulating their surface receptors, microglial cells are activated, produce pro‐inflammatory molecules, and induce inflammation at the stimulus site, which is prominent in most neurodegenerative disorders, including AD.^12^ Although these inflammatory responses are from the M1 type of activated microglia, which is the inflammatory type,^13^ the other type is M2, which is the anti‐inflammatory type that modulates the immune system's response, produces anti‐inflammatory molecules and neurotrophic factors and has enhanced phagocytosis ability.^13^

It has been shown that decreasing inflammation and inhibiting inflammatory cells with anti‐inflammatory drugs in AD does not improve the cognitive function of the patients.^14^ On the contrary, in‐vivo studies have shown that polarisation of microglia to M2 type ameliorates cognitive impairments and decreases neuronal apoptosis.^15^ This suggests that inhibition of the M1 type alone is insufficient, and the M2 type microglia population should also be increased. Several studies indicate that specific preconditioning approaches induce M1/M2 polarisation of microglial cells toward M2 type.^16^, ^17^, ^18^, ^19^ Additionally, preconditioning methods have been used in brain injuries and neurodegenerative disorders, including AD.^20^, ^21^ This implies that preconditioning of the microglial cells may be beneficial in treating and preventing AD. Here we review preconditioning approaches of the microglial cells as a possible option for preventing AD development.

## 2 MICROGLIA ACTIVATION AND NEUROINFLAMMATION

**FIGURE 1:**
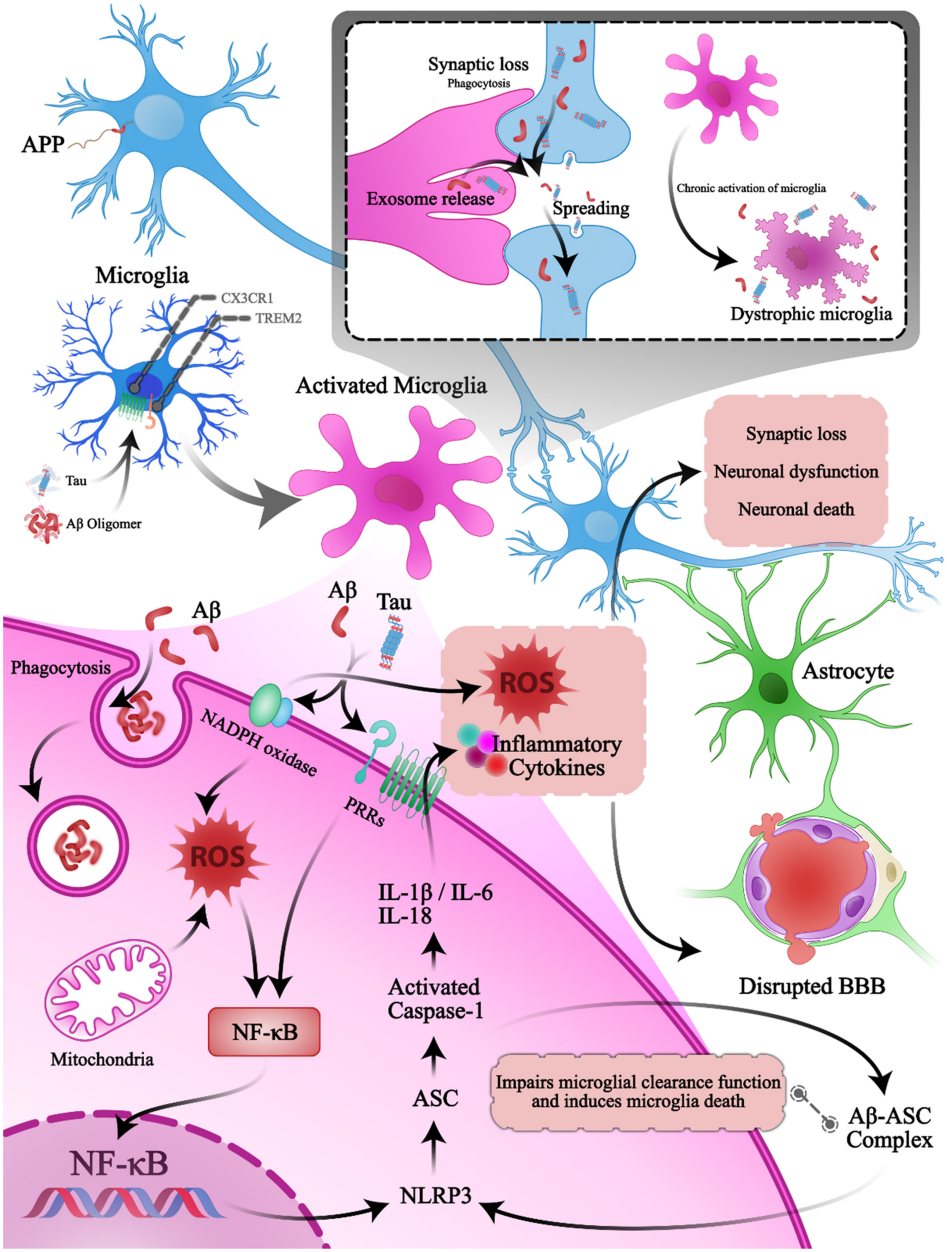
Role of microglia in molecular pathways involved in AD. One of the roles of microglia is the phagocytosis of Aβ. Tau and Aβ are recognized by microglial PRRs and activate inflammatory cascades via NF‐κB/NLRP3 inflammasome activation. Following NLRP3 inflammasome activation, ASC is released into the cytosol, which subsequently causes Caspase‐1 activation and IL‐1β/IL‐18 secretion. Extracellular ROS disrupt BBB, synaptic loss, neuronal dysfunction, and eventually neuronal death. In this process, mitochondrial dysfunction can also produce ROS and activate NF‐κB. Activation of NADPH oxidase, following Aβ/tau recognition, induces intra‐ and extracellular ROS production. Microglial cells also spread tau and Aβ between neurons by releasing tau/Aβ containing exosomes. Furthermore, microglial cell phagocytoses the neurons' synapses that contain tau and Aβ aggregates, which results in a synaptic loss. Chronic activation results in microglial dystrophy, and as a consequence, the phagocytosis function of these cells is impaired, which causes sustained inflammation. APP, amyloid precursor protein; ASC, apoptosis‐associated speck‐like protein containing a C‐terminal caspase recruitment domain; Aβ, amyloid‐βeta; BBB, blood–brain barrier; NLRP3, NF‐κB activation and consequent assembly of the pyrin domain‐containing 3; PRRs, Pattern recognition receptors; ROS, reactive oxygen species.

Recruitment of immune cells, especially microglia/astroglia, and inflammatory mediators' production in the brain trigger inflammatory responses in AD.^22^ There is a positive feedback loop between the aberrant activation of microglia and neuroinflammation. Activated microglia induces neuroinflammation through increasing pro‐inflammatory cytokine and reactive oxygen species (ROS) production.^22^, ^23^ Microglial‐derived pro‐inflammatory cytokines and ROS can cause neurotoxicity (synaptic loss, neuronal dysfunction and neuronal apoptosis), contributing to cognitive impairments^24^ (Figure 1).

Microglial activation can be triggered by microenvironment changes such as systemic inflammation, promote cytotoxic and inflammatory responses, and reduce homeostatic microglia.^25^ Chronic pathological stimuli confrontation in the later stages of the disease causes morphological and immunological alteration of the activated microglia toward the M1 and dystrophic types.^26^, ^27^, ^28^, ^29^ In a normal inflammatory state in CNS, after the elimination of pathologic stimulus, activated microglial cells are switched from M1 to M2 type with the higher phagocytic ability and modulation of inflammation function. This switch hinders excessive inflammation and other CNS dysfunction.^30^ Conversely, in AD, the pathologic factors, which are misfolded Aβ and tau proteins, are not eliminated through the function of microglial cells and are more aggregated in the brain as the disease progresses, acting as repetitive stimuli for microglia activation. This type of aberrantly activated microglia is called primed microglia, which is neurotoxic and less protective and has a lower ability to switch into a naïve state. Therefore, microglial cells are not changed into anti‐inflammatory phenotype and cause sustained and chronic inflammation leading to disease progression.^30^, ^31^, ^32^, ^33^

In addition, as mentioned earlier activated microglia can induces neuroinflammation through ROS production. Studies have reported an amplification loop between the activation of microglia and ROS production during neuroinflammation, causing blood disruption and neurotoxicity. Microglia‐induced ROS production can increase fibrinogen infiltration into the CNS by increasing blood–brain barrier (BBB) permeability, producing more microglia activation and additive ROS.^34^, ^35^, ^36^, ^37^ Furthermore, ROS‐induced mitochondrial dysfunction and higher NADPH oxidase production in microglia are involved in the direct neurotoxicity effect of microglia via extracellular ROS production. Also, it induces intracellular ROS production in microglia, promoting pro‐inflammatory and neurotoxic cytokine leads production and activating NF‐κB, JNK and JAK/STAT pathways^38^ (Figure 1).

As mentioned, microglial cells play critical roles in endothelial cell destruction and BBB impairment by activating detrimental pathways and producing pro‐inflammatory substances.^39^, ^40^, ^41^ Microglia‐induced local and systemic inflammation causes BBB breakdown and promotes vascular dementia (VD) and AD pathogenesis.^42^, ^43^ Due to BBB impairment, microglial cells induce aberrant transportation of inflammatory mediators and infiltration of trans‐endothelial immune cells into the brain resulting in sustained neuroinflammation.^35^, ^41^ The BBB disruption spreads peripheral inflammation into the CNS mainly due to direct contact with inflamed endothelial cells and neurons.^44^, ^45^ Increased BBB vulnerability to inflammation may be due to Aβ‐induced alteration in endothelial function and increased oxidative stress in endothelial cells.^43^

Another vicious cycle that is triggered by activation of microglia is between microglia activation and Aβ/tau neuropathology. Both soluble oligomers and vascular deposit forms of Aβ and tau aggregation induce microglia activation and proinflammatory gene expression through binding to microglial cell surface receptors such as CX3CR1.^46^, ^47^, ^48^, ^49^ In addition, tau pathology enhances neuroinflammation by activating the NF‐κB/cGAS/STING pathway, leading to microglial activation and BBB impairment.^8^, ^50^ NF‐κB is one of the essential components of this cycle, which regulates inflammatory responses. NF‐κB can be activated by microglia surface receptors such as CX3CL1/CX3CR1 axis activation.^51^ Recognition of Aβ via microglial pattern recognition receptors (PRRs) activates inflammatory cascades through NF‐κB activation and consequent assembly of the pyrin domain‐containing 3 (NLRP3) inflammasome.^52^ The NLRP3 inflammasome plays a vital role in microglia‐related neuroinflammation and neuronal death via the NLRP3/caspase‐1 activation and subsequent IL‐1 and IL‐18 secretion.^22^ NLRP3 inflammasome reduces Aβ phagocytosis leading to the formation of Aβ plaques. NLRP3 inflammasome regulates tau kinases and phosphatases, which induce tau hyperphosphorylation. Moreover, it is involved in Aβ‐induced tau phosphorylation, or else, fibrillar Aβ and tau monomers and oligomers can activate the NLRP3 inflammasome.^53^, ^54^, ^55^

## 3 THE ROLE OF MICROGLIA IN AD

Despite the role of microglial cells in the pathophysiology of AD, the imperative role of microglia in neuroprotection and maintaining synaptic functions proved,^11^ such as surveying neurons and synapses, detecting and resolving abnormalities by secreting inflammatory molecules and phagocytosis, maintaining synaptic plasticity, and provide a neuronal communication network.^11^, ^12^, ^56^, ^57^ In the early stages of AD, microglial cells are beneficial for preventing AD's progression by enhancing the clearance of Aβ.^56^ It could be suggested that the excessive activation of these cells would result in increased phagocytosis, and eventually, it would overcome amyloid plaque aggregation. However, studies have reported that excessive activation of microglial cells results in decreased phagocytosis ability of these cells as inflammation proceeds.^58^, ^59^ Impaired microglial phagocytosis due to astrocytic protection,^56^ prolonged microglia activation, and aberrant inflammation are detrimental at later stages.^58^, ^59^ In addition, excessive activation of microglial cells during AD results in reverse outcomes, including synaptic dysfunction, neural destruction, BBB impairment, and increased inflammatory state in the CNS, resulting in behavioural and cognitive dysfunction^39^, ^40^, ^41^, ^60^, ^61^, ^62^ (Figure 1). Additionally, chronic Aβ‐induced activation of microglial cells contributes to the dysfunction of these cells due to metabolic defects.^58^, ^63^ Studies have reported a dystrophic type of microglial cells in AD pathogenesis that could be induced by excessive microglial activation, which has impaired phagocytosis and results in sustained elevated inflammation.^12^, ^64^, ^65^

## 4 MICROGLIA AND TAU PATHOLOGY

Although much progress has been made in recent years regarding the role of microglia in AD, the precise mechanisms underlying microglia‐related tauopathies still need to be determined. Studies have shown that microglia may have a principal role in tauopathy in AD, which is highly associated with functional deficits in AD.^66^ There is a feed‐forward loop between tau pathology and microglial activation. Microglia can exacerbate tau pathology; otherwise, tau can cause microglial dysfunction.^59^ Microgliosis and early synaptic loss have been reported in tauopathies as early manifestations causing cognitive decline.^67^, ^68^, ^69^ Microglia participate in tau‐induced synaptic dysfunction via aberrant microglial phagocytic activity in the synapses of neurons with tau aggregations resulting in synaptic loss.^70^ Following the phagocytosis, microglia secrete tau seeds inducing other tau aggregation and promoting tau spreading to other neurons.^70^, ^71^ Microglia‐associated synaptic spreading of tau is mainly via secreting tau‐containing exosomes, leading to propagation throughout the brain.^72^, ^73^, ^74^ So, suppressing exosome secretion from microglia can reduce the tau burden in AD.^72^, ^75^ Other possible mechanisms of tau secretion include synaptic vesicle exocytosis,^76^ secretion in plasma membrane‐shed ectosomes,^77^ and directly through the plasma membrane in a non‐vesicular manner.^78^, ^79^, ^80^, ^81^ Although unclear, tau released from neurons may benefit the extracellular space under physiological conditions without cell death.^76^, ^82^ Increased tau secretion to the extracellular space upon neuronal degeneration is toxic for microglial cells and neurons, possibly through a direct effect on them.^83^, ^84^ Excessive pre‐synaptic extracellular tau secretion contributes to synaptic dysfunction in AD,^85^ and post‐synaptic neurons can take up the secreted tau, resulting in trans‐synaptic tau spreading.^76^

Besides increased tau secretion, decreased tau clearance will also affect extracellular tau levels. Microglia were also shown to play a pivotal role in extracellular tau clearance through phagocytosis.^86^ Studies have suggested that infiltrated macrophages and microglial cells can internalize and degrade extracellular tau in a neuroinflammatory environment.^87^, ^88^ Following tau phagocytosis by microglial cells in the later stages of AD, these cells become hypofunctional and express a senescence‐like phenotype, leading to the decreased ability of tau clearance and exacerbation of neurodegeneration.^70^ It has been shown that intra‐ and extracellular tau induces abnormal microglial proliferation and activation, leading to pathological neuronal and synaptic loss.^60^, ^61^ On the contrary, abnormal microglial activation is possibly associated with impaired tau phagocytosis capacity resulting in tau aggregation and consequent axonal and dendritic damage in later stages of AD.^62^, ^69^, ^84^, ^89^ The secretome of microglia can alter the structure and function of physiological form and promote the formation of tau misfolded monomer, which results in tau and NFT aggregation inside the cells.^50^ Studies have also shown that altered morphology and dystrophy of microglia are associated with hyperphosphorylation and accumulation of tau, pathologic tau spreading, and consequent neurodegeneration in AD.^90^, ^91^, ^92^ Significant microglial degeneration detected in the hippocampus of AD patients is mainly due to soluble intra−/extracellular phosphorylated tau toxicity after phagocytosis.^29^ It has been demonstrated that removing senescent astrocytes and microglial cells reduced tau hyperphosphorylation and tau‐induced neurodegeneration.^93^ So, targeting tau‐induced microglial activation could impede the progression of cognitive decline.

## 5 MICROGLIA AND AΒ PATHOLOGY

Studies have reported the association between Aβ plaques and activated microglial cells.^94^ Upon Aβ plaque formation, microglial cells are recruited and activated.^95^ Aβ aggregates, taken up by activated microglia, are accumulated in lysosomes due to impaired clearance capacity. Intracellular accumulated Aβ induces microglial cell death, and as a consequence, accumulated Aβ is released into the extracellular space and induces Aβ plaque formation and expansion. Eventually, this vicious cycle leads to additional microglial recruitment/activation and Aβ plaque formation/expansion.^96^ Microglia are also involved in Aβ‐induced neurotoxicity. Following Aβ‐induced microglial activation, microglial cells release pro‐inflammatory secretions resulting in neuronal death.^97^ Activated microglia‐derived microvesicles (MVs) containing Aβ are also neurotoxic and are involved in forming neurotoxic Aβ species in the extracellular environment. Furthermore, MVs can contribute to delivering neurotoxic species to other neurons after Aβ phagocytosis.^98^ It has been shown that the expression of Aβ‐binding receptors and phagocytic capacity of Aβ are decreased following aberrant and chronic activation of microglia in the later stages of AD. Meanwhile, the capability to produce pro‐inflammatory cytokines is still maintained—all of these lead to the aggregation of Aβ in late‐stage AD.^99^

Aβ causes mitochondrial toxicity in microglia by targeting the F0F1 ATP synthase leading to ROS generation and dysregulation of microglial cells' functions. In this situation, impaired extracellular Aβ phagocytic activity occurs. It also causes pro‐inflammatory cytokine release via activation of the NF‐κB pathway and NLRP3 inflammasome.^100^ Furthermore, microglial cells also induce apoptosis‐associated speck‐like protein containing a C‐terminal caspase recruitment domain (ASC). Specks released into the intercellular space via NLRP3 inflammasome activation induce Aβ seeds secretion resulting in Aβ aggregation and spreading.^101^, ^102^ Seed‐induced Aβ deposits are involved in neurogenesis impairments and cell death induction, leading to cognitive decline and memory impairment. It has been proved that activated microglial phagocytic activity inhibits Aβ seeding and its consequences.^103^ ASC‐Aβ composites cause additional inflammasome activation. Intra‐ and extracellular ASC‐Aβ impair Aβ clearance function of microglial cells, leading to microglial pyroptotic death and consequent other ASC release^104^ (Figure 1).

Microglial cells might also be involved in Aβ–associated tau bioactivity and spreading.^105^ On the other hand, studies have shown that microglia might also contribute to delaying Aβ‐induced pathological tau propagation through TREM2 activity.^106^ So, TREM2 activity plays an essential role in the phagocytic clearance of amyloid.

## 6 MICROGLIA‐ASSOCIATED TARGETS FOR AD TREATMENT

Recently, genome‐wide association studies (GWAS) considered that many genetic risk factors for AD are robustly associated with microglial cells.^107^, ^108^ Thus, it is presumed that microglia‐associated neuroinflammation is not only the consequence of AD but also a determining factor in developing and provoking AD. In this section, we will review the current suggested microglia‐associated targets and pathways, their potential roles in AD pathogenesis, and the potential challenges of each target.

### 6.1 CD33

It was shown in 2011 that polymorphism of CD33, a member of the sialic acid‐binding immunoglobulin‐like lectin (singles) family, is involved in AD development and pathology. CD33 in the brain, mainly expressed by microglial cells, suppresses Aβ phagocytosis.^109^ Moreover, an increased expression of CD33 that promotes plaque pathology has been shown in the cortex of patients with AD. It has been demonstrated that CD33‐deficient mice had lesser amyloid pathology, suggesting that CD33 has a crucial role in amyloid pathology.^109^ Another study suggested that the increased expression of CD33 in the peripheral system plays a crucial role in the pathogenesis of AD.^110^ Given this variety of data, targeting CD33 and its potential action site may indicate a promising approach for treating AD. P22, as a subtype‐selective sialic acid mimetic, had increased Aβ uptake when it was attached to microparticles in a CD33‐dependent manner.^111^ Furthermore, many antibodies against CD33 can be a potential drug to treat AD. In particular, lintuzumab, a recent monoclonal antibody for treating acute myelogenous leukaemia, would be a possible candidate for defeating AD.^112^ There are several considerations and potential challenges to address before targeting CD33 as a therapeutic approach for AD. These include determining whether CD33 is directly linked to the disease, establishing the feasibility of manipulating its function or levels, and overcoming the difficulty of reversing the damage that may have accumulated over a lifetime. Additionally, the development of small‐molecule or antibody‐based approaches to target CD33 may face obstacles such as poor BBB penetration, low brain exposure, and potential safety consequences due to peripheral CD33 targeting.^113^ Taking it all together, CD33 remains a candidate for potential AD therapeutic targets with special challenges that need future experiments.

### 6.2 APOE

The ε4 isoform of the Apolipoprotein E (APOE) is the most potent risk factor for AD and a common genetic variant that has a crucial role in CNS inflammatory response.^114^, ^115^ In the brain, APOE is expressed by astrocytes, microglial cells, and neurons and has crucial role in lipid transport and synaptic homestasis. It is shown that the interaction between APOE and Aβ plaques is regulated by microglia.^116^ Moreover, APOE has a different response according to its isoforms. The *ε4* isoform raises AD risk, whereas the *ε2* isoform diminishes AD risk. However, APOE in the presence of Aβ can reduce microglia response to Aβ plaque, initiating an inflammatory cascade. This controversy could indicate the complex interactions between microglia, glial cells, and APOE.^117^ APOE may be involved in AD through Aβ accumulation and clearance as modulating microglia function and cytokine release. Existing approaches for targeting APOE as a therapeutic intervention for AD can be grouped into three main classifications^118^: first, increasing APOE quantity and degree of lipidation that could enhance lipid transport and modulate AD‐related pathways,^119^ second, targeting APOE structural properties and its interaction with Aβ with focus on the use of small molecules to disrupt the abnormal interaction between the amino‐terminal and carboxy‐terminal domains of APOE4,^120^ and third, targeting APOE receptors such as LDLR and LRP1, which are known to play a role in Aβ clearance in the brain. By upregulating the expression of these receptors, it may be possible to enhance Aβ clearance and reduce Aβ‐associated pathology, thus potentially alleviating the disease's progression.^121^

In targeting proteins with essential physiological functions like APOE, a key challenge lies in minimising potential adverse effects on APOE‐dependent brain homeostasis and systemic physiology. While some individuals with APOE deficiency may not exhibit significant neurocognitive deficits, the long‐term consequences on brain physiology, particularly in aging and AD development, remain unknown, necessitating close monitoring of APOE modulation effects at various stages of disease progression and consideration of potential impacts on peripheral lipid metabolism.

### 6.3 TREM2

Triggering receptor expressed on myeloid cells 2 (TREM2) is an immunoreceptor expressed by macrophages, dendritic cells, and brain microglia, and it is one of the remarkably well‐studied genes associated with AD.^122^ Microglia cells' selective expression of TREM2 in the brain connects these cells to the pathogenicity of neurodegenerative disease.^123^

GWAS has identified mutation R47H, among TREM2 variants, as a significant risk factor of AD that diminishes microglia responses to amyloid pathology in the brain.^124^ Moreover, TREM2 expression was increased in plaque‐associated microglia,^125^ and modulating TREM2 expression can reprogram microglia activity.^126^

Most studies reveal that TREM2 can promote a microglia‐mediated response to Aβ; however, there are some controversies in this regard.^127^ While TREM2 is essential for microglial activation and clustering in all tested Aβ models, its impact on Aβ plaque burden varies between models. TREM2 deficiency increased plaque burden in the 5XFAD^128^ but unexpectedly decreased amyloid burden in the APPPS1‐21 model,^129^ possibly due to a shift toward anti‐inflammatory processes in the TREM2‐deficient myeloid cells model. Despite all these controversies about TREM2 implication in the microglia‐mediated inflammatory response to Aβ deposition, they all agree that TREM2 modulates the inflammatory process and function of microglia cells, including proliferation and Aβ phagocytosis. Moreover, Colonna and colleagues suggested that impaired TREM2 function diverts the mammalian rapamycin signals involved in energy and anabolic metabolism.^122^ Increasing autophagic‐like vesicles in microglia cells causes the death of cells and microglia loss. Thus, microglia cannot appropriately eradicate Aβ plaques due to inadequate energy supply, dysfunction, and astroglial responses.^56^, ^130^, ^131^ Conversely, CD33 can diminish Aβ phagocytosis, increasing the risk of AD.^109^, ^132^ TREM2 and APOE are linked, suggesting that TREM2 plays a role in APOE expression and signalling.^133^

In short, TREM2 is a promising therapeutic target if the pharmacologic modulations could upregulate the expression or activity of this immunoreceptor. Another proposed method is antibodies stabilising these proteins on the surface of cells by blocking their cleavage enzymes. There is much to know and investigate possible ways to oppose AD effects by utilising TREM2 as a therapeutic target.

### 6.4 P2X7/NLPR3

ATP‐gated ion channels, P2x receptors, are purinergic receptors expressed by microglial cells and are sensitive to extracellular ATP, usually increased due to tissue damage or distress. P2X7 has received extensive attention as it is a key mediator for NLRP3 (nucleotide‐binding oligomerisation domain, leucine‐rich repeat, and pyrin domain‐containing inflammasome complex 3) activation leading to the excretion of pro‐inflammatory cytokines such as IL‐1β and IL‐18.^134^ There are increased amounts of P2X7R in the brain of patients with AD.^135^, ^136^ Moreover, rats that received Aβ peptide in their hippocampus had elevated P2X7R expression.^135^ P2X7R deficiency decreases the microglial‐inflammatory response stimulated by ATP and Aβ. Notably, the P2X7–NLRP3 pathway is an exciting target for drug discovery as some P2X7 antagonists are clinically utilized, and NLRP3 inhibitors are in progress.^53^, ^137^, ^138^

### 6.5 Microglial calcium signalling

Calcium signalling is a crucial second messenger in the majority of cell types and also a pivotal mediator for microglia functions.^139^ An increase in microglia's intracellular calcium level is necessary for them to release cytokines and chemokines.^140^ Although the role of calcium signalling in neurons and the pathogenesis of AD has been shown before, it is still unclear how calcium signalling in microglia is associated with the pathogenesis of AD.^141^ Combs et al., in a study in 1999, demonstrated that the co‐culturing of microglia with Aβ25‐35 peptide increases intracellular calcium transiently. The release of this Ca^2+^ activates microglia and leads to neuroinflammation. AD mouse models have an altered level of P2YR‐associated calcium signalling in microglia around the plaque. However, the Ca^2+^ level in distant microglia was similar to matched control groups.^142^ It has been shown by McLarnon and colleagues that calcium signalling is dysregulated in microglia cells of AD patients. In other words, their microglia have higher basal Ca^2+^ levels but a lower amplitude and longer‐lasting ATP‐induced calcium response compared to the control group.^19^ Elevated levels of intracellular Ca^2+^ activate an intermediate‐conductance potassium channel named K_Ca_3.1 and subsequently lead to k^+^ efflux. This efflux fortifies Ca^2+^ influx via Ca^2+^ release‐activated calcium (CRAC) channels, developing Ca^2+^ accumulation.^25^ Voltage‐independent K_Ca_3.1‐like Ca^2+^‐activated K^+^ currents inhibitors, charybdotoxin, and clotrimazole inhibit microglial oxidative bursts. As calcium signalling has a decisive role in many functions of microglia, its association with the pathogenesis of AD is an exciting option for future investigations.

### 6.6 Extracellular vesicles

Extracellular vehicles (EVs) are a heterogeneous family of lipid‐bound vesicles, including exosomes and MVs, facilitating intercellular communication. Microglia cells secrete EVs and MVs to mediate their inflammatory signalling. In Aβ pathology, microglial cells that have phagocytosed Aβ secrete EVs containing Aβ and phosphorylated tau.^72^, ^143^ It has been shown that microglial exosomes are crucial in seeding tau pathology in the nervous system. In an article by Polanco and colleagues, the injection of EVs extracted from tau transgenic mouse brains into wild‐type mice increases the propagation of tau pathology.^144^

EVs demonstrate significant potential as a therapeutic tool for neurodegenerative diseases due to their ability to efficiently target the brain and penetrate the BBB. Specifically, stem cell‐derived EVs exhibit neuroprotective and immunomodulatory properties. In AD, EVs overexpressing neprilysin can reduce Aβ plaque deposition and protect neurons against Aβ‐beta‐induced oxidative stress and synaptic damage.^145^, ^146^, ^147^ This neuroprotection can be mediated by delivering specific cargo, such as miRNAs to neurons, inhibiting BACE1 expression, and activating the Wnt/β‐catenin pathway.^148^ Emerging engineering strategies are being developed to further explore the specific targeting of EVs, including modification of their parent cells for tailored delivery and targeting.^149^

## 7 MICROGLIAL CELLS PRECONDITIONING IN THE TREATMENT OF ALZHEIMER'S DISEASE

Preconditioning is a process in which specific cells or tissues are exposed to a less toxic or original stimulus to produce a protective response when facing a condition or disorder.^150^ Murray and colleagues first proposed preconditioning in an experimental model in dogs by inducing brief heart ischemia followed by reperfusion.^151^ The results of this study encouraged scientists to apply the precondition of ischemia (ischemic preconditioning; IPC) and other stimuli to other animal models and humans. Preconditioning methods have also been used in neurodegenerative disorders. Hypoxia preconditioning has been reported to prevent AD‐like phenotype in rats via mitochondrial adaptations.^20^ However, preconditioning methods are not limited to IPC and hypoxia, and other stimuli can be used as preconditioning agents and stimuli to help manage neurodegenerative disorders, especially AD. Here, we discuss microglial cells preconditioning with different stimuli and their effect on AD.

### 7.1 TLRs

**FIGURE 2:**
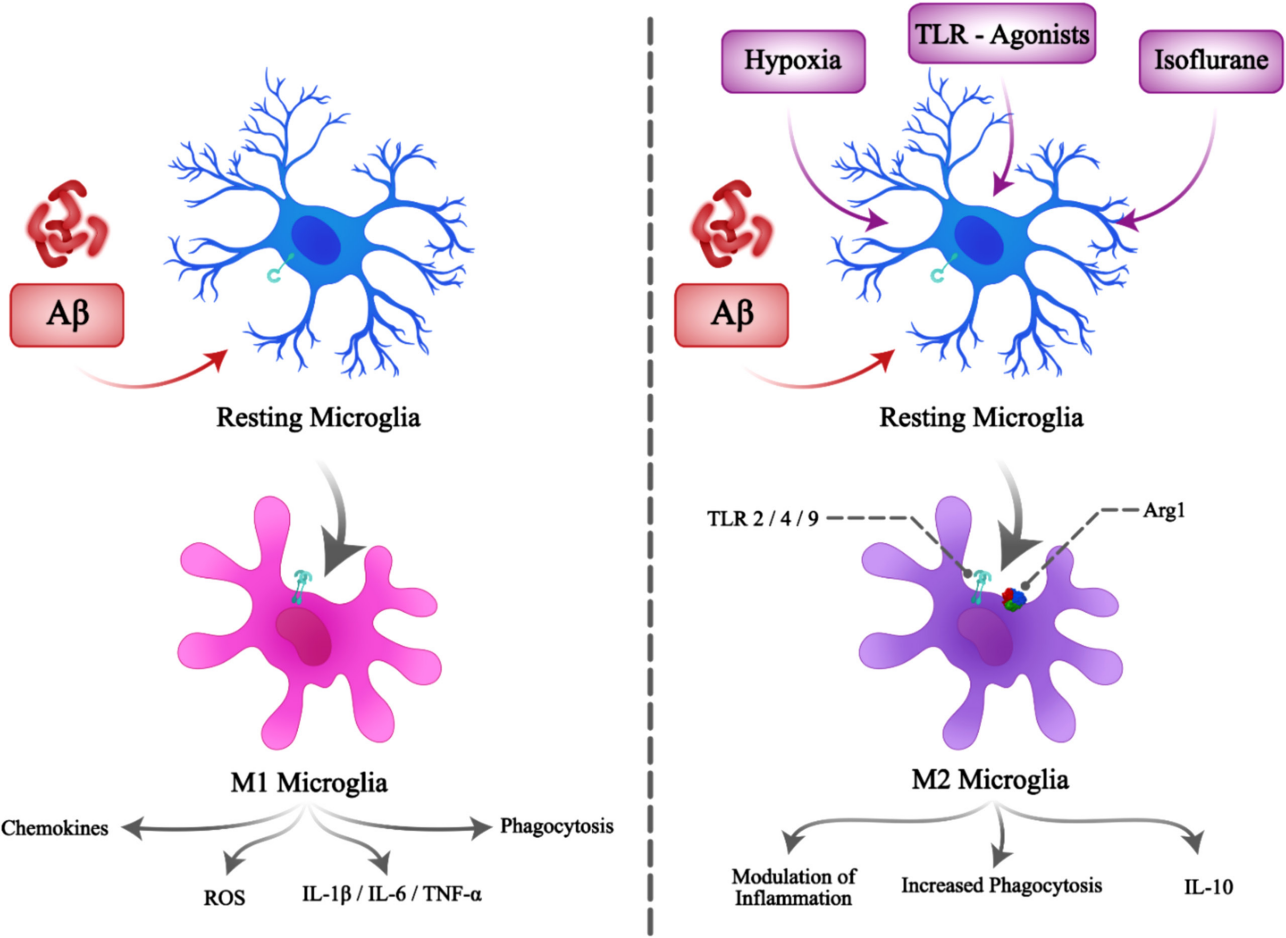
Microglial cell preconditioning in AD. Aβ induces the transformation of resting microglial cells into M1‐type microglial cells. This results in the activation of phagocytosis, ROS production, and release of inflammatory cytokines and chemokines, which induce inflammation and further injury. On the contrary, preconditioning microglial cells prior to Aβ exposure transforms them into M2 types. It decreases inflammation by releasing anti‐inflammatory cytokines such as IL‐10 while maintaining their phagocytosis capability. Aβ, amyloid‐βeta; ROS, Reactive oxygen species; TLR, toll‐like receptor.

Toll‐like receptors (TLRs) are members of a family which recognize various pathogens in the innate immune system and bring further immune responses, called PRRs. PRRs recognize multiple molecules on the surface of pathogens called pathogen‐associated molecular patterns (PAMPs). Although expressed in different immune cells like monocytes, macrophages, microglia, and dendritic cells, they are also present in other cells such as neurons, astrocytes, and fibroblasts. There are 10 types of TLRs in humans, and TLR1‐9 is present both in humans and murine.^152^ Microglia express all TLRs on their surface with different levels.^153^ However, TLRs are highly expressed in activated microglia. Various data demonstrate the role of TLRs in mediating the production of inflammatory and cytotoxic molecules from microglia, implying their prominent contribution to AD pathogenesis.^154^ On the contrary, TLRs have been shown to mediate microglial phagocytosis function in the presence of pathogens, including Aβ, and are, therefore, crucial in improving AD.^155^, ^156^ Thus, the actions of TLRs are like a two‐edged sword. Among TLRs, three are thought to most participate in microglia‐mediated Aβ phagocytosis, including TLR2, TLR4 (and its co‐receptor CD14) and TLR9. However, TLR2 and TLR4 also mediate the production of cytotoxic molecules from microglia.^152^ Numerous studies have used the preconditioning method to evaluate whether it helps ameliorate AD. Pourbadie et al. assessed the effect of microglia preconditioning with LPS/MPL (TLR4 agonists) and Pam3cys (TLR2 agonist) on improving cognitive and memory conditions, levels of inflammatory and anti‐inflammatory cytokines, M2 type microglial cell marker (arginase 1) and Aβ uptake and the receptor involved FPR2.^17^ Pretreatment with TLR2 and TLR4 agonists, both in vitro and in vivo, increased FPR2 and Aβ uptake, increased arginase‐1 marker and anti‐inflammatory cytokines like IL‐10 and TGF‐β, implicating conversion of microglia to M2 type, which is demonstrated to diminish Aβ load in the brain and produce anti‐inflammatory cytokines^18^ (Figure 2). They showed the subsequent behavioural, cognitive, and memory improvement and preservation of electrophysiological properties in a rat model of AD. Similarly, Michaud et al. studied the effect of pretreatment with TLR4 agonist MPL.^157^ The results were similar, and microglial cells' phagocytosis function was elevated without inducing pro‐inflammatory cytokines. Also, in another experiment, microglial cells were pretreated with low doses of LPS and MPL.^16^ The results of both in‐vitro and in‐vivo experiments were increased production of anti‐inflammatory cytokines (IFN‐β) and diminished secretion of TGF‐α. The author mentioned that IFN‐β has neuroprotective effects by transforming microglial cells to M2 type and decreasing IL‐6, IL‐8 and TNF‐α. Tahara et al. reported the same outcomes in the pretreatment of microglial cells with TLR2, TLR4 and TLR9 agonists. Aβ phagocytosis is triggered by the TLR agonists.^156^ Furthermore, it has also been shown that microglial cell preconditioning with TLR2 and TLR4 agonists results in decreased pro‐inflammatory and increased anti‐inflammatory cytokines in response to pilocarpine.^158^ Consistently, pretreatment with TLRs agonists in other conditions like ischemia, trauma, and epilepsy also resulted in neuroprotection and reduced brain damage.^158^, ^159^, ^160^, ^161^ TLR9 agonist (CpG) is also utilized in microglial cells. In vitro, studies demonstrated that CpG preconditioning significantly elevated Aβ phagocytosis without inducing inflammatory and neurotoxic molecules. Moreover, preconditioned microglial cells produced antioxidant enzymes such as HO‐1 and MMP‐9 after applying Aβ. In‐vivo part of this study showed cognitive and behavioural improvement alongside decreased Aβ load in the brain after intraventricular injection of CpG.^162^ Treatment with CpG in AD mouse models decreased cerebral vascular Aβ loads without causing any microhemorrhages and improved mice cognition and behaviours. Anti‐inflammatory cytokines were also increased.^163^, ^164^, ^165^ Furthermore, pretreatment of microglial cells with TLR2 agonist, pam3cys, has been shown to increase the production of IFN‐β both in‐vitro and in‐vivo, with possible subsequent AD improvement.^166^

Most of the studies mentioned above stated that low doses of Aβ did not induce the activation of microglial cells. In contrast, high doses activated and polarized microglial cells to the M1 type, producing pro‐inflammatory cytokines and neurotoxic molecules. This implies that microglial cells are not activated in the early stages of AD, where Aβ concentrations are low. Their activation in response to high concentrations of Aβ is detrimental to neurons by producing pro‐inflammatory cytokines. However, these studies demonstrated that microglial cells preconditioned with TLRs agonists not only transformed into M2 types in response to Aβ but were activated in low Aβ concentrations, which might prevent AD from progressing in the early stages.

Some studies have shown possible mechanisms of protective effects of microglial preconditioning. Sangaran et al. showed that LPS‐induced microglia produce inflammatory cytokines, whereas LPS preconditioning of microglia results in increased anti‐inflammatory cytokines. They reported that LPS induction activates TLR‐4 and leads to NF‐κB translocation into the nucleus and subsequent pro‐inflammatory cytokine production. However, pre‐activation of TLR‐4 by low‐dose LPS resulted in the inhibition of NF‐κB translocation and increased levels of anti‐inflammatory cytokines, including IL‐10,^167^ which indicates the M2 microglia activation. Additionally, it has been shown that cytosolic Ca^2+^‐mediated internalisation of TLR‐4 is necessary for the activation of TRIF/IRF‐3 pathway.^168^ Similarly, TLR‐2 preconditioning of microglia induces anti‐inflammatory cytokines production and a neuroprotective state of microglia via TRIF/IRF‐3 pathway.^166^

Clinical implications of the preconditioning of microglia with TLRs for the management of AD are limited. However, clinical studies have used TLR agonists for other conditions.^169^ For instance, MPLA is an FDA‐approved TLR‐4 agonist that can be used as an adjuvant in viral vaccines.^169^, ^170^ Also, both agonists and antagonists of TLR‐4 have been evaluated in the form of adjuvants and drugs for different conditions, including cancers, inflammation and immune disorders.^169^ In addition, TRL‐2 antagonist OPN‐305 (anti‐TLR‐2 antibody) has been used to prevent reperfusion injury after renal transplantation in a phase I clinical trial.^171^ Another example of TLRs in clinical studies is the evaluation of inhaled TLR‐9 agonist AZD1419 for the treatment of asthma in a phase I clinical trial.^172^ Moreover, EMD 1201081, a TLR‐9 agonist, was evaluated in combination with immunotherapy for head and neck malignancies.^173^ These data suggest that preconditioning with TRLs is clinically applicable and can be used in other conditions, including AD.

### 7.2 Hypoxia

Studies have considered ischemia as a predisposing factor for the long‐term development of AD via the activation of glial cells, including microglial cells.^174^, ^175^, ^176^ Although, in preconditioning, much less hypoxic stress is induced to evaluate the outcomes and effects. In an experiment, hypoxia preconditioning in rat models caused increased production of anti‐inflammatory cytokines such as IL‐10 and TGF‐β and elevated M2‐type microglia markers (Arg1) levels, indicating microglia phenotype alteration^177^ (Figure 2). Additionally, Hypoxic preconditioning inhibits M1 polarisation of microglia by reducing NLRP3 formation via NF‐κB pathway inhibition.^19^ Also, in‐vitro hypoxia‐preconditioned cultured microglial cells produced diminished nitrite, IL‐1β, and TNF‐α levels. COX‐2 gene expression was also repressed, and overall, these results suggest decreased microglial cells' activity.^178^ Additionally, Correia et al. reported the preventive effects of hypoxia on AD‐like phenotype in rats via mitochondrial adaptation resulting from improved respiratory chain function and increased mitochondrial biogenesis and DNA (mtDNA) content.^20^ Since mitochondrial dysfunction^179^ and mtDNA damage^180^ have been suggested to have an important role in the development of AD, targeting mitochondria as a preventive strategy has been proposed in AD.^181^ Cells maintain the population of functional mitochondria through mitochondrial autophagy, fission‐fusion, and biogenesis processes.^182^ These are also considered processes of mitochondrial adaptation to stresses.^183^ Prolonged hypoxia can cause mitochondrial adaptation in cultured cells by inducing mitochondrial autophagy and decreasing ROS production.^184^ Furthermore, mitochondrial adaptation causes the prevention and improvement of neurodegenerative disorders.^181^, ^185^

In clinical assessments, an experiment on several elderly patients was carried out to evaluate the effect of intermittent hypoxia‐hyperoxia training (IHHT) on AD development from its early state. The results revealed that IHHT could halt AD progression.^186^ Yet, the effects on microglial cells were not assessed. As implications for clinical investigations, cell transplantation, such as stem cell transplantation, has been shown to have promising therapeutic effects in in‐vivo studies for AD^187^ and other neurological conditions, including seizures.^188^ Furthermore, preconditioned stem cell transplantation has been shown to increase the efficacy of these cells in AD rat models.^189^ Additionally, stem cells have been preconditioned with hypoxia and transplanted in the brains of animals to improve the effectiveness of these cells.^190^, ^191^ This approach can be applied to microglia as well. It has been shown that human CSF contains a population of microglial cells that are detected by surface markers.^192^ Therefore, these cells can be extracted from each patient who has preconditioning processes and be transplanted into in the brain. Nevertheless, to our knowledge, there are no clinical studies for cell transplantation in the brain and these approaches can be implicated in clinical applications for the management of AD.

### 7.3 Isoflurane

Isoflurane is a volatile anaesthetic used in inducing generalized anaesthesia. The neurotoxic or neuroprotective effect of isoflurane is under debate as it demonstrates both effects. Although microglial cell preconditioning with isoflurane and assessment of subsequent effects on preventing AD development have not yet been studied, numerous studies evaluated the effect of isoflurane preconditioning (IP) on microglial cells' inflammatory response. Sun et al. showed IP of microglial cells causes a decrease in TLR4 expression and inhibits M1 microglia activation by inhibiting NF‐κB pathway signalling and subsequent reduction in TNF‐α and IL‐1 production (Figure 2). Isoflurane, 2% for 30 min, was applied in this experiment.^193^ On the contrary, the IP of H4 human neuroglioma cells with isoflurane 1.4% for 2 h evoked cellular apoptosis and increased Aβ deposition.^194^ Xu et al. suggested that this dual effect of IP is due to the dose and time of isoflurane exposure. They demonstrated that low doses of IP for a shorter period diminish Aβ‐induced H4 human neuroglioma cells apoptosis. On the contrary, high doses with a longer time of exposure increase apoptosis.^195^ Although these pieces of evidence are not precisely regarded to our goal in this review because of the cell lines used in their experiments, they provide good evidence for future studies to assess the effect of IP on microglial cells to prevent AD development.

## 8 CONCLUSION AND PROSPECTS

Despite the progress in finding new treatment approaches for AD, no drug has improved the disease, and some limited choices are available for patients to delay the progression of the disease and the development of its symptoms. Current evidence suggests that the preconditioning of microglial cells for preventing AD development is promising. To the best of our knowledge, other possible types of this approach for AD have not yet been evaluated; therefore, other different consequently further experiments are necessary to experiment with new preconditioning techniques and assess their effectiveness. Moreover, the existing evidence for preconditioning in AD in‐vivo studies and the translatability of these approaches to humans should be evaluated. This could be achieved using cell therapy and regenerative methods combined with these preconditioning approaches. Another hypothesis could be microglial preconditioning without isolation of these cells by application of exsosomes inside the brain with different techniques like nanotechnology as the preconditioning approach. Additionally, it has been shown that preconditioning with natural products or minerals can successfully prevent or reduce the outcome of serious conditions. For instance, long term (9 months) dietary sodium nitrate preconditioning in female rats has been shown to reduce the myocardial infarct size following ischemia–reperfusion injury.^196^ Furthermore, several natural compounds have been found to act as agonists or antagonists of TLRs^197^ that can be used in a long‐term period as dietary supplements and their effectiveness on AD initiation, progression, or management can be assessed. As for hypoxia preconditioning, it has been used in clinical practice for other conditions, including rehabilitation for COVID‐19.^198^ This strategy can also be suggested for AD. Also, hypoxic exercise can be used to clinically assess hypoxic preconditioning's effect on AD.^199^ Therefore, preconditioning approaches can be applied in clinical practice for the prevention of AD. Overall, preconditioning could be a new opportunity for the potential treatment of AD, and future studies are necessary to improve this approach so it can be used in the clinic.

## AUTHOR CONTRIBUTIONS

**Younes Yassaghi:** Conceptualization (equal); writing – original draft (equal); writing – review and editing (equal). **Yasaman Nazerian:** Conceptualization (equal); writing – original draft (equal); writing – review and editing (equal). **Mobina Ghasemi:** Conceptualization (equal); writing – original draft (equal); writing – review and editing (equal). **Amirhossein Nazerian:** Conceptualization (equal); writing – original draft (equal); writing – review and editing (equal). **Fatemeh Sayehmiri:** Conceptualization (equal); supervision (equal); writing – review and editing (equal). **George Perry:** Conceptualization (equal); writing – review and editing (equal). **Hamid Gholami Pourbadie:** Conceptualization (equal); supervision (equal); writing – review and editing (equal).

## FUNDING INFORMATION

This research received no external funding.

## CONFLICT OF INTEREST STATEMENT

The authors confirm that there are no conflicts of interest.

## Data Availability

Not applicable.
